# Association between oxidative balance score and kidney stones: data from the national health and nutrition examination survey (NHANES)

**DOI:** 10.1186/s12882-024-03607-w

**Published:** 2024-06-03

**Authors:** Rundong Song, Ke Wu, Minghai Ma, Lu Wang, Yunzhong Jiang, Jianpeng Li, Jinhai Fan

**Affiliations:** 1 Department of Urology, The First Affiliated Hospital of Xi’an Jiaotong University, Xi’an, Shaanxi 710061 China; 2 The Second Affiliated Hospital of Xi’an Jiaotong University, 710004 Xi’an, Shaanxi China

**Keywords:** Kidney stones, Oxidative balance score, Reactive oxygen species, National health and nutrition examination survey, Restricted cubic spline regression analysis

## Abstract

**Purpose:**

Some studies have found that the pathological formation of kidney stones is closely related to injury and inflammatory response. Behaviors such as dietary composition, physical activity, obesity and smoking can all affect the body’s oxidative stress levels. In order to evaluate the effects of various diets and lifestyles on the body’s oxidative and antioxidant systems, an oxidative balance score was developed. To investigate whether the OBS is associated with the development of kidney stones.

**Methods:**

Data were taken from the National Health and Nutrition Examination Survey (NHANES) from 2007–2018, followed by retrospective observational studies. The association between kidney stones and OBS was analyzed using survey-weighted logistic regression by adjusting for demographics, laboratory tests, and medical comorbidity covariates. The oxidative balance score is calculated by screening 16 nutrients and 4 lifestyle factors, including 5 prooxidants and 15 antioxidants, based on prior information about the relationship between oxidation levels in the body and nutrients or lifestyle factors.

**Results:**

A total of 26,786 adult participants were included in the study, of which 2,578, or 9.62%, had a history of nephrolithiasis. Weighted logistic regression analysis found an association between OBS and kidney stones. In the fully tuned model, i.e., model 3, the highest quartile array of OBS was associated with the lowest quartile array of OBS (OR = 0.73 (0.57, 0.92)) with the risk of kidney stone (*p* = 0.01), and was statistically significant and remained relatively stable in each model. At the same time, the trend test in the model is also statistically significant. With the increase of OBS, the OR value of kidney stones generally tends to decrease.

**Conclusions:**

There is an inverse correlation between OBS and kidney stone disease. At the same time, higher OBS suggests that antioxidant exposure is greater than pro-oxidative exposure in diet and lifestyle, and is associated with a lower risk of kidney stones

## Introduction

Kidney stones are a common disease in urology, and its incidence has been increasing in recent years [1]. In the United States, there is a disproportionate increase in the incidence and prevalence of kidney stone disease in women and in black and Hispanic individuals [2]. The disease has a long course and is prone to recurrence, causing serious damage to kidney function and imposing a huge economic burden on patients and society [3, 4]. Previous studies have shown that gender, race, age, lifestyle, and diet are important factors in stone formation [5, 6]. The formation of stones is not yet fully understood, and there are various theories, including renal calcified plaque, supersaturated crystals, stone matrix, crystal-inhibiting substances, and heterogeneity-promoting nucleation theory. In most patients, the underlying etiology is thought to be multifactorial, including environmental, dietary, hormonal, and genetic components [7]. In addition, abnormalities in the body’s metabolism, obstruction of the urinary tract, infections, foreign bodies, and the use of medications are common causes of stone formation. Focusing on and addressing these issues can reduce stone formation and recurrence.

Previous studies have shown that the pathology of kidney stone formation is closely related to injury and inflammatory responses where reactive oxygen species (ROS) -induced oxidative stress is essential [8, 9]. ROS originate predominantly in injured mitochondria, and calcium salt crystals significantly damage epithelial cell mitochondria and exacerbate the inflammatory response [10]. In physiological conditions, there is a balance between oxidants and antioxidant systems. When ROS production exceeds the scavenging capacity of the antioxidant response system, large amounts of proteins are oxidized, and lipid peroxidation occurs. Excessive production of ROS by epithelial cells promotes crystal aggregation, growth, and adhesion, ultimately leading to stone formation [11].

However, the effect of a single certain factor on the body’s oxidative and antioxidant systems is limited, and various dietary components, physical activity, obesity, and behaviors such as smoking all affect the body’s oxidative stress levels. Therefore, to assess the effects of various diets and lifestyles on the body’s oxidative and antioxidant systems, the Oxidative Balance Score (OBS) was developed to reflect the overall balance of dietary and lifestyle-promoted oxidant and antioxidant exposure [12]. In general, higher OBS indicates that antioxidants are superior to pro-oxidants. Previous studies have found that OBS is negatively associated with various diseases, including digestive, respiratory, cardiovascular, and type II diabetes. However, no studies have evaluated the relationship between kidney stones and OBS. We hypothesized that OBS might be associated with an increased risk of developing kidney stones, and to answer this question. We examined the relationship between OBS and kidney stones in a nationally representative survey controlling for various known risk factors for kidney stones. This study aimed to assess the association between OBS and kidney stones in U.S. adults using data from the National Health and Nutrition Examination Survey (NHANES).

## Material and methods

### Study population

The National Center for Health Statistics (NCHS) annually surveys randomly selected, non-institutionalized U.S. civilians. Certain participant subgroups, such as the Hispanic, black, and elderly populations, are intentionally oversampled to reflect the U.S. population’s demographic composition accurately. The survey assessed the demographics, socioeconomic status, and health status of a nationally representative sample of U.S. residents. We analyzed respondents who completed relevant kidney status questionnaires over 6 NHANES cycles (2007–2018), including questions about kidney stones. Subjects who answered "refused," "missing," or "do not know" to questions assessing kidney stones were excluded. This study is a retrospective observational study.

### Definition of kidney stones

Kidney stones were defined as an affirmative response to "Have you ever had kidney stones?" from a single survey question (KIQ026).

### Oxidative balance score (OBS)

OBS was calculated based on a priori information on the relationship between O.S. and nutrients or lifestyle factors by screening 16 nutrients and 4 lifestyle factors, including 5 pro-oxidants and 15 antioxidants. Dietary intakes of 16 nutrients, including dietary fiber, carotenoids, riboflavin, niacin, vitamin B6, total folate, vitamin B12, vitamin C, vitamin E, calcium, magnesium, zinc, copper, selenium, total Dietary intakes of 16 nutrients, including total fat and iron, were obtained from the first dietary review interview.4 Lifestyle factors were physical activity, body mass index (BMI), alcohol consumption, and smoking, with the extent of smoking indicated by cotinine. Of these, total fat, iron, BMI, alcohol consumption, and smoking were considered pro-oxidants, and the rest were considered antioxidants. Referring to the method of calculating OBS, alcohol consumption was categorized into 3 groups, heavy drinkers (women ≥ 15 g / d, men ≥ 30 g / d), non-heavy drinkers (women 0 ∼ 15 g / d, men 0 ∼ 30 g / d), and non-drinkers, which were assigned scores of 0, 1, and 2, respectively. After that, the other components were grouped by gender and then divided into 3 groups by tertiles, where antioxidants were assigned a value of 0 ∼ 2, and pro-oxidants were assigned a value of 2 ∼ 0 in groups 1 ∼ 3 [12]. The higher the OBS scores, the more significant the antioxidant exposure. Subjects with ≥ 16 complete data for each of the 20 OBS components were selected for this study. For OBS with missing components, we assigned a score of 0 corresponding to the missing component, either antioxidant or pro-oxidant.

### Assessment of covariates

In our study, covariates were certain factors previously shown or hypothesized to be associated with kidney stones or OBS, including sociodemographic variables, indicators of inflammation, diet quality, and comorbidities. Sociodemographic variables included age, sex (male, female), race (Mexican American, non-Hispanic black, non-Hispanic white, other Hispanic, other race - including multiracial), education (less than high school, high school, greater than high school), marital status (divorced/separated/widowed, married/living with a partner, never married), and the ratio of household income to poverty level (< 1.3, 1.3 ∼ 3.5, > 3.5). Inflammatory indicators reflected their status by the number of leukocytes, neutrophils, lymphocytes, and monocytes. Overall dietary quality was assessed using the 2015 version of the Healthy Eating Index (HEI) and total energy intake [13]. Comorbidities included hypertension, cardiovascular disease, diabetes mellitus, arthritis, and hyperlipidemia, with hypertension and diabetes mellitus diagnosed by index measurements, medication use, and self-report, and the remaining comorbidities identified by self-report.

### Statistical analysis

All statistical analyses were performed using R version 4.3.0 (R Foundation for Statistical Computing, Vienna, Austria; http://www.r-project.org), and differences were considered statistically significant at a *P* value < 0.05. Weighting was performed using the NHANES-recommended weight selection and calculation method. Characterization of sociodemographic variables, inflammatory indicators, diet quality, comorbidities, and oxidative balance scores for the prevalence or absence of kidney stones was performed for the weighted data. In the baseline characterization, continuous variables were expressed as weighted means (standard errors), and categorical variables were expressed as sample sizes (weighted percentages). To test for differences in variable characteristics between OBS groups (quartiles), differences in weighted means for continuous variables were analyzed using ANOVA, and differences in weighted percentages for categorical variables were analyzed using the Rao - Scott χ 2 test to characterize the total population. A weighted logistic regression model explored the association between OBS and kidney stones. To validate the correlation between OBS and kidney stones and to explore the possibility of a nonlinear relationship between OBS and kidney stones, the continuous variable OBS was transformed into a categorical variable by quartiles, and a trend *P* value was calculated. A total of 4 models were used in this study, and the crude model did not adjust for any potential confounders. Model 1 adjusted for age, sex, race/ethnicity, marital status, poverty income ratio, and education. Model 2 further adjusted for white blood cells, neutrophils, lymphocytes, monocyte, Healthy Eating Index, and Total energy intake. Model 3 was additionally adjusted for five comorbidities. The OBS was categorized into dietary OBS and lifestyle OBS, and their associations with kidney stones were discussed separately. Finally, restricted cubic spline (RCS) regression was used to verify the relationship between kidney stones and OBS.

## Results

### Baseline characteristics

**Fig. 1:**
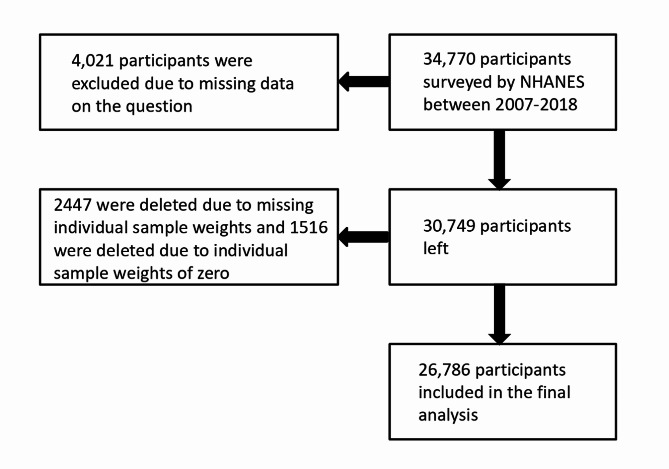
Participant selection criteria

Thirty-four thousand seven hundred seventy survey respondents who responded to the kidney stone questionnaire were first identified from NHANES. Four Thousand Twenty One respondents were removed due to missing data from the questions (Fig. 1). When applying survey weights, 2,447 were removed due to missing individual sample weights, and 1,516 were removed due to zero individual sample weights (Fig. 1). Baseline demographic information is detailed in Table 1.9.62% of the survey respondents had a history of kidney stones. The mean age (± S.E.) of the population was 48.17 ± 0.25, and those with kidney stones were somewhat older, with a significant correlation (*p* < 0.0001). Of the total population, 47.64% were males and 52.36% were females, and a higher percentage of males in the population with the disease remained significantly correlated (*p* < 0.0001).42.82% self-identified as non-Hispanic white, 21.71% self-identified as non-Hispanic black, 14.41% self-identified as Mexican American, 10.17% self-identified as other Hispanic, 10.17% self-identified as other Hispanic, and 10.17% self-identified as other Hispanic and identified as Other Hispanic, and 10.88% self-identified as Other, with a significant correlation (*p* < 0.0001) when comparing participants with and without a history of kidney stones. For different marital statuses, there was a significant correlation with having kidney stones (*p* < 0.0001). There was no significant correlation between the presence of kidney stones and other sociodemographic variables such as poverty-income ratio and educational attainment. When comparing the presence or absence of kidney stones with our indicators of interest, OBS, diet-related OBS, and lifestyle-related OBS, all were found to be significantly correlated (*p* < 0.0001, *p* < 0.001, *p* < 0.0001). There was no correlation between the presence of kidney stones and total energy intake, and there was a significant correlation with Healthy Eating Index (*p* < 0.0001). In comparing their levels with the inflammation-related indicators, leukocytes, neutrophils, lymphocytes, and monocytes were all correlated. When comparing subjects with and without a history of kidney stones, a significant correlation was found between patients with stones and several comorbidities (Table 1), all with *p*-values less than 0.0001.

**Table 1:** The baseline characteristics by kidney stones: National Health and Nutrition Examination Survey 2007–2018

	Total	History of kidney stones		
		No	Yes	*p* value
Age(year), Mean (S.E)	48.17(0.25)	47.50(0.27)	54.31(0.41)	**< 0.0001**
Sex, n (%)				**< 0.0001**
Female	14025(52.36)	12873(53.47)	1152(45.55)	
Male	12761(47.64)	11335(46.53)	1426(54.45)	
Race/ethnicity, n (%)				**< 0.0001**
Mexican American	3859(14.41)	3540(8.39)	319(5.87)	
Non-Hispanic Black	5816(21.71)	5470(11.87)	346(6.03)	
Non-Hispanic White	11471(42.82)	10023(66.29)	1448(77.73)	
Other Hispanic	2725(10.17)	2447(5.64)	278(4.96)	
Other Race - Including Multi-Racial	2915(10.88)	2728(7.80)	187(5.41)	
Poverty income ratio, n (%)				0.2
1.3–3.5	9236(37.84)	8308(34.89)	928(37.28)	
Less than 1.3	7663(31.39)	6937(21.87)	726(20.36)	
More than 3.5	7511(30.77)	6795(43.24)	716(42.37)	
Marital status, n (%)				**< 0.0001**
Divorced/separated/widowed	5956(22.25)	5281(18.37)	675(21.44)	
Married/living with a partner	16006(59.78)	14352(62.25)	1654(68.75)	
Never married	4812(17.97)	4565(19.37)	247(9.81)	
Education, n (%)				0.96
Less than high school	6105(22.81)	5502(14.76)	603(14.53)	
high school	6128(22.9)	5535(23.11)	593(23.27)	
More than high school	14527(54.29)	13146(62.13)	1381(62.20)	
OBS, Mean (S.E)	21.11(0.12)	21.22(0.12)	20.14(0.23)	**< 0.0001**
Dietary OBS, Mean (S.E)	16.98(0.10)	17.06(0.10)	16.25(0.21)	**< 0.001**
Lifestyle OBS, Mean (S.E)	4.13(0.02)	4.16(0.03)	3.89(0.04)	**< 0.0001**
Total energy intake, Mean (S.E)	2131.68(8.89)	2132.78(9.46)	2121.50(29.60)	0.72
Healthy Eating Index, Mean (S.E)	51.35(0.24)	51.53(0.24)	49.64(0.38)	**< 0.0001**
White blood cell, Mean (S.E)	7.31(0.03)	7.29(0.03)	7.47(0.07)	**0.01**
Neutrophils, Mean (S.E)	4.34(0.02)	4.32(0.02)	4.51(0.06)	**< 0.001**
Lymphocyte, Mean (S.E)	2.16(0.01)	2.16(0.01)	2.11(0.02)	**0.05**
Monocyte, Mean (S.E)	0.57(0.00)	0.57(0.00)	0.58(0.01)	**0.01**
Hypertension, n (%)				**< 0.0001**
No	15126(56.47)	14031(63.12)	1095(46.93)	
Yes	11659(43.53)	10176(36.88)	1483(53.07)	
Diabetes mellitus, n (%)				**< 0.0001**
No	21340(80.58)	19544(86.62)	1796(74.67)	
Yes	5144(19.42)	4374(13.38)	770(25.33)	
Arthritis, n (%)				**< 0.0001**
No	19113(71.49)	17632(74.33)	1481(59.56)	
Yes	7621(28.51)	6530(25.67)	1091(40.44)	
Cardiovascular disease, n (%)				**< 0.0001**
No	24008(89.64)	21914(92.51)	2094(84.17)	
Yes	2775(10.36)	2292(7.49)	483(15.83)	
Hyperlipidemia, n (%)				**< 0.0001**
No	8009(29.9)	7437(31.36)	572(23.26)	
Yes	18775(70.1)	16769(68.64)	2006(76.74)	

### Baseline characteristics of individuals grouped by OBS quartile

Compared to the lowest OBS quartile, participants in the highest OBS quartile group had the following characteristics: younger age, non-Hispanic white or other race, higher wealth, married or partnered, higher education level, higher HEI, higher total energy intake, lower white blood cell levels, lower neutrophil levels, lower lymphocyte levels, and lower monocyte levels. The prevalence of kidney stones and several of their comorbidities, including hypertension, diabetes, cardiovascular disease, arthritis, and hyperlipidemia, decreased progressively with increasing OBS. Differences in gender between OBS quartile groups were not statistically significant. (Table 2)

**Table 2:** The baseline characteristics by quartiles of the OBS: National Health and Nutrition Examination Survey 2007–2018

Characteristic	Total	Q1,(3, 15)	Q2,(15,20]	Q3,(20,26]	Q4,(26,37]	*p* value
	n = 26,836	n = 7635	n = 5829	n = 7324	n = 5998	
Age(year),Mean (S.E)	48.17(0.25)	49.09(0.36)	48.80(0.39)	47.99(0.35)	47.01(0.40)	**< 0.001**
Sex, n (%)						0.06
Female	14025(52.36)	3807(51.27)	3037(51.85)	3957(54.40)	3224(52.85)	
Male	12761(47.64)	3828(48.73)	2792(48.15)	3367(45.60)	2774(47.15)	
Race/ethnicity, n (%)						**< 0.0001**
Mexican American	3859(14.41)	960(7.64)	848(8.38)	1130(8.68)	921(7.83)	
Non-Hispanic Black	5816(21.71)	2362(18.38)	1288(11.83)	1349(9.62)	817(6.25)	
Non-Hispanic White	11471(42.82)	2936(61.20)	2515(67.16)	3196(68.37)	2824(72.23)	
Other Hispanic	2725(10.17)	774(6.13)	598(5.62)	774(5.45)	579(5.17)	
Other Race	2915(10.88)	603(6.65)	580(7.00)	875(7.88)	857(8.53)	
Poverty income ratio, n (%)						**< 0.0001**
1.3–3.5	9236(37.84)	2669(38.40)	2108(37.14)	2500(34.91)	1959(30.80)	
Less than 1.3	7663(31.39)	2825(32.07)	1702(22.49)	1848(18.86)	1288(14.92)	
More than 3.5	7511(30.77)	1399(29.52)	1507(40.37)	2325(46.22)	2280(54.28)	
Marital status, n (%)						**< 0.0001**
Divorced/separated/widowed	5956(22.25)	2051(23.42)	1324(19.44)	1519(17.30)	1062(15.21)	
Married/living with a partner	16006(59.78)	4132(55.85)	3465(62.79)	4551(65.25)	3858(66.82)	
Never married	4812(17.97)	1446(20.73)	1040(17.77)	1250(17.45)	1076(17.97)	
Education, n (%)						**< 0.0001**
Less than high school	6105(22.81)	2367(22.35)	1428(16.62)	1418(12.69)	892(8.51)	
High school	6128(22.9)	2062(29.14)	1389(26.28)	1622(22.15)	1055(16.15)	
More than high school	14527(54.29)	3200(48.51)	3003(57.11)	4276(65.16)	4048(75.33)	
Total energy intake, Mean (S.E)	2131.68(8.89)	1531.73(12.04)	1959.75(16.15)	2251.73(14.14)	2687.18(21.60)	**< 0.0001**
Healthy Eating Index, Mean (S.E)	51.35(0.24)	44.86(0.24)	49.20(0.28)	52.31(0.29)	57.95(0.31)	**< 0.0001**
White blood cell, Mean (S.E)	7.31(0.03)	7.60(0.05)	7.47(0.05)	7.28(0.04)	6.96(0.04)	**< 0.0001**
Neutrophils, Mean (S.E)	4.34(0.02)	4.53(0.04)	4.44(0.03)	4.32(0.03)	4.10(0.04)	**< 0.0001**
Lymphocyte, Mean (S.E)	2.16(0.01)	2.22(0.02)	2.20(0.03)	2.15(0.02)	2.08(0.01)	**< 0.0001**
Monocyte, Mean (S.E)	0.57(0.00)	0.58(0.00)	0.58(0.01)	0.57(0.00)	0.55(0.00)	**< 0.0001**
Kidney stones, n (%)						**< 0.0001**
No	24208(90.38)	6762(88.04)	5267(90.52)	6659(90.52)	5520(91.72)	
Yes	2578(9.62)	873(11.96)	562(9.48)	665(9.48)	478(8.28)	
Hypertension, n (%)						**< 0.0001**
No	15126(56.47)	3692(55.28)	3200(58.73)	4305(62.32)	3929(68.69)	
Yes	11659(43.53)	3943(44.72)	2628(41.27)	3019(37.68)	2069(31.31)	
Diabetes mellitus, n (%)						**< 0.0001**
No	21340(80.58)	5698(80.92)	4560(83.66)	5937(85.83)	5145(90.63)	
Yes	5144(19.42)	1894(19.08)	1217(16.34)	1290(14.17)	743(9.37)	
Arthritis, n (%)						**< 0.0001**
No	19113(71.49)	5043(68.71)	4152(72.96)	5333(72.87)	4585(76.68)	
Yes	7621(28.51)	2578(31.29)	1665(27.04)	1978(27.13)	1400(23.32)	
Cardiovascular disease,n (%)						**< 0.0001**
No	24008(89.64)	6483(87.54)	5191(91.00)	6715(92.98)	5619(94.65)	
Yes	2775(10.36)	1150(12.46)	638(9.00)	608(7.02)	379(5.35)	
Hyperlipidemia, n (%)						**< 0.0001**
No	8009(29.9)	2090(28.03)	1625(28.13)	2198(30.07)	2096(35.41)	
Yes	18775(70.1)	5545(71.97)	4203(71.87)	5125(69.93)	3902(64.59)	

### Association between OBS and kidney stones

Weighted logistic regression analysis revealed an association between OBS and kidney stones, as detailed in Table 3. A total of 4 models were used, and the crude model did not adjust for any potential confounders. Model 1 adjusted for age, sex, race/ethnicity, marital status, poverty income ratio, and education. Model 2 was further adjusted for white blood cells, neutrophils, lymphocytes, monocyte, Healthy Eating Index, and Total energy intake. Model 3 was additionally adjusted for 5 comorbidities. In Model 3, the highest quartile group of OBS compared with the lowest quartile group of OBS (OR = 0.73 (0.57,0.92)) was correlated with the risk of kidney stone prevalence (*p* = 0.01). It was statistically significant, remaining relatively stable across models. Similarly compared to the lowest quartile group of OBS, the second and third quartiles of OBS were correlated with the risk of kidney stone prevalence, and both were statistically significant (Q2: OR = 0.76 (0.61,0.93), *p* = 0.01; Q3: OR = 0.81 (0.66, 0.98), *p* < 0.03). Also, the test for trends in the model was statistically significant.

**Table 3:** Weighted logistic regression analysis models showing the associations between OBS and kidney stones

OBS	Crude model		Model 1		Model 2			Model 3	
	OR 95%CI	P	OR 95%CI	P	OR 95%CI	P	OR 95%CI		P
Q1	Ref		Ref		Ref		Ref		
Q2	0.77(0.64,0.92)	0.01	0.74(0.61,0.89)	0.002	0.75(0.61,0.91)	0.01	0.76(0.61,0.93)		0.01
Q3	0.77(0.65,0.91)	0.002	0.76(0.64,0.91)	0.003	0.80(0.66,0.96)	0.02	0.81(0.66,0.98)		0.03
Q4	0.66(0.56,0.80)	< 0.0001	0.63(0.51,0.77)	< 0.0001	0.68(0.54,0.87)	0.002	0.73(0.57,0.92)		0.01
p for trend		< 0.0001		< 0.0001		0.003			0.01

Crude model: Unadjusted model

Model 1: Adjusted for age, sex, race/ethnicity, marital status, poverty income ratio, education

Model 2: Additionally adjusted for white blood cell, neutrophils, lymphocyte, monocyte, Healthy Eating Index, Total energy intake

Model 3: Additionally adjusted for hypertension, diabetes mellitus, arthritis, cardiovascular disease, hyperlipidemia

### Associations of dietary OBS and lifestyle OBS with kidney stones

Weighted logistic regression analyses revealed the presence or absence of associations between dietary OBS and kidney stones, as detailed in Table 4. 4 models were still used, and the crude model was not adjusted for any potential confounders. Model 1 adjusted for age, sex, race/ethnicity, marital status, poverty income ratio, and education. Model 2 was further adjusted for white blood cells, neutrophils, lymphocytes, monocyte, Healthy Eating Index, and Total energy intake. Model 3 was additionally adjusted for 5 comorbidities. In Model 3, the highest quartile of OBS compared to the lowest quartile of OBS (OR = 0.80 (0.61,1.05)) may not be correlated with the risk of prevalence of kidney stones (*p* = 0.1).

**Table 4:** Weighted logistic regression analysis models showing the associations between Dietary OBS and kidney stones

Dietary OBS	Crude model		Model 1		Model 2		Model 3	
	OR 95%CI	P	OR 95%CI	P	OR 95%CI	P	OR 95%CI	P
Q1	Ref		Ref		Ref		Ref	
Q2	0.87(0.75,1.01)	0.08	0.85(0.72,1.00)	0.05	0.87(0.72,1.06)	0.17	0.87(0.72,1.05)	0.15
Q3	0.80(0.68,0.95)	0.01	0.79(0.66,0.96)	0.02	0.85(0.69,1.04)	0.12	0.84(0.68,1.04)	0.10
Q4	0.74(0.61,0.89)	0.001	0.71(0.58,0.86)	< 0.001	0.79(0.60,1.04)	0.09	0.80(0.61,1.05)	0.10
p for trend		< 0.001		< 0.001		0.07		0.08

Crude model: Unadjusted model

Model 1: Adjusted for age, sex, race/ethnicity, marital status, poverty income ratio, education

Model 2: Additionally adjusted for white blood cell, neutrophils, lymphocyte, monocyte, Healthy Eating Index, Total energy intake

Model 3: Additionally adjusted for hypertension, diabetes mellitus, arthritis, cardiovascular disease, hyperlipidemia

Similarly, compared to the lowest quartile group of OBS, the second and third quartile groups of OBS may not correlate with the risk of kidney stone prevalence. Also, the test for trends in the model was not statistically significant. Weighted logistic regression analysis found an association between lifestyle OBS and kidney stones, as detailed in Table 5. The model used was the same as before. In model 3, the highest quartile group of OBS compared to the lowest quartile group of OBS (OR = 0.74 (0.62,0.90)) was correlated with the risk of kidney stone prevalence (*p* = 0.002). It was statistically significant, remaining relatively stable across models. However, there may be no correlation between the second and third quartiles of OBS and the risk of kidney stone prevalence compared to the lowest quartile of OBS. However, the test for trends in the models was statistically significant.

**Table 5:** Weighted logistic regression analysis models showing the associations between Lifestyle OBS and kidney stones

Lifestyle OBS	Crude model		Model 1		Model 2		Model 3	
	OR 95%CI	P	OR 95%CI	P	OR 95%CI	P	OR 95%CI	P
Q1	Ref		Ref		Ref		Ref	
Q2	0.88(0.74,1.04)	0.14	0.83(0.69,1.00)	0.05	0.88(0.73,1.06)	0.18	0.96(0.79,1.16)	0.66
Q3	0.84(0.72,0.98)	0.02	0.82(0.69,0.96)	0.02	0.86(0.72,1.02)	0.08	0.96(0.80,1.15)	0.65
Q4	0.60(0.51,0.70)	< 0.0001	0.57(0.48,0.67)	< 0.0001	0.63(0.53,0.76)	< 0.0001	0.74(0.62,0.90)	0.002
p for trend		< 0.0001		< 0.0001		< 0.0001		0.01

Crude model: Unadjusted model

Model 1: Adjusted for age, sex, race/ethnicity, marital status, poverty income ratio, education

Model 2: Additionally adjusted for white blood cell, neutrophils, lymphocyte,monocyte, Healthy Eating Index, Total energy intake

Model 3: Additionally adjusted for hypertension, diabetes mellitus, arthritis, cardiovascular disease, hyperlipidemia

### Restricted cubic spline regression analysis

**Fig. 2:**
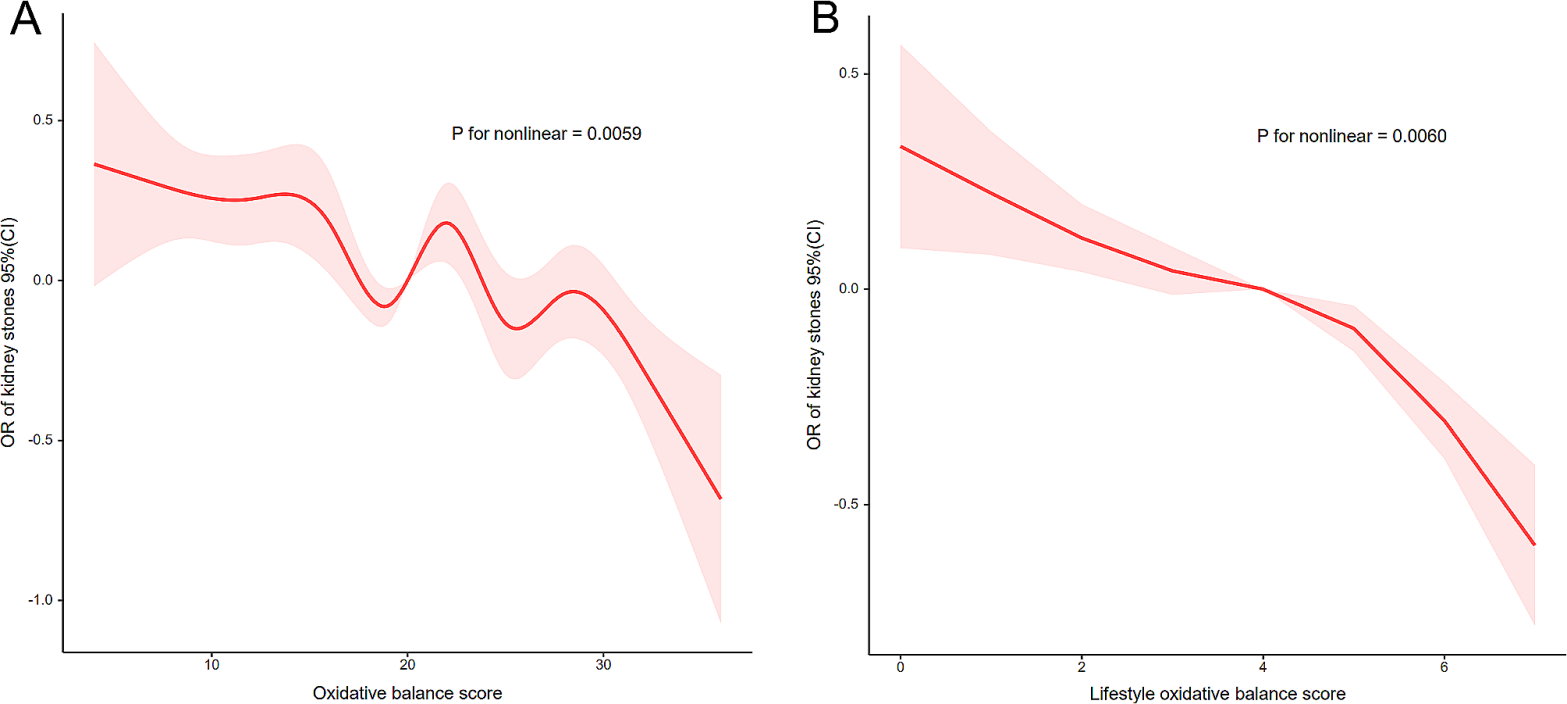
Analysis of restricted cubic spline regression

In restricted cubic spline regression, adjusting for different covariates, we found a significant nonlinear relationship between OBS and kidney stones (p for nonlinear < 0.0059, Fig. 2A). Figure 2A shows an overall trend of decreasing OR for kidney stones with increasing OBS, but between OBS values of 20 and 23, there is again a trend of increasing OR, which is worth pondering and discussing. There was a nonlinear negative correlation between lifestyle OBS and kidney stones (p for nonlinear < 0.0060, Fig. 2B). Figure 2B shows an overall trend of decreasing OR for kidney stones with increasing lifestyle OBS. Despite the differences in the results of the nonlinear analysis of the restricted triple spline, the overall trends of the dependent and independent variables were generally consistent across the plots.

## Discussion

To discuss the relationship between kidney stones and oxidative balance scores, we analyzed data from about 26,786 participants from a nationally representative survey population in the United States. We found a correlation between kidney stones and OBS. Even after adjusting for sociodemographic variables (age, sex, race, education, marital status, and ratio of household income to poverty), indicators of inflammation (number of leukocytes, neutrophils, lymphocytes, and monocytes), the Healthy Eating Index (HEI) and total energy intake to assess overall dietary quality, and covariates for comorbidities including hypertension, cardiovascular disease, diabetes mellitus, arthritis, and hyperlipidemia. This association persisted after the variables.

There are several possible drivers behind our finding of a lower prevalence of kidney stones in the presence of high OBS levels compared to participants with low OBS levels. One possible explanation is the difference in ROS levels in the body of subjects with different oxidative balance scores. Higher OBS levels imply that antioxidants are superior to pro-oxidants in the organism [12]. Therefore, subjects with high levels of OBS have lower levels of ROS in their bodies, and it has been shown that ROS-induced oxidative stress is essential in the pathogenesis of kidney stones,8,9 so this reason may have contributed to the low prevalence of kidney stones in participants with high levels of OBS. Other researchers have proposed that ROS production and the progression of oxidative stress may be a common pathophysiologic basis for kidney stones and other metabolic diseases [14]. These findings can provide us with a research idea and direction to study kidney stones and other diseases, which can further investigate the relationship between reactive oxygen species and kidney stones and explore their molecular biological mechanisms.

Because there are no studies to examine the direct relationship between oxidative balance scores and the prevalence of kidney stones, there are studies in previously published articles that have involved examining the relationship between obesity and kidney stones and have found that obesity can independently lead to kidney stones in the absence of metabolic abnormalities and insulin resistance [15]. In some studies, obesity is also characterized by chronic low-grade inflammation and permanently increased oxidative stress [16]. In obese animals or humans, adipose tissue is characterized by increased local and systemic production of pro-inflammatory adipocytokines that induce ROS production [17]. Elevated ROS leads to important changes in adipose tissue, which promote a systemic low-grade inflammatory response with adverse effects throughout the body [18]. Summarizing the results of these studies, the idea can be put forward that obese people may produce more reactive oxygen species and are, therefore, more likely to suffer from kidney stone disease. The OBS can be used as a good evaluative criterion to determine the oxidative and antioxidant status that the participants are in, which can be used to predict the development of kidney stones through the OBS and also to change the level of the OBS by adjusting the diet and the lifestyle in to prevent the occurrence of kidney stones. Other researchers have found an association between dietary intake of riboflavin and thiamine and kidney stones, with higher riboflavin intake negatively associated with kidney stones [19]. In our study, riboflavin was considered an antioxidant in the OBS calculations. The higher the intake, the higher the score, so it is consistent with the findings of our study. One study revealed a negative association between the level of dietary selenium intake and the risk of kidney stones in the U.S. population, especially for young adults (< 50 years old), men, and those who are overweight/obese (BMI ≥ 25.0) [20], which is also consistent with the findings of our study. The next step could be modeling to predict the incidence of kidney stones in the population, and the molecular biology of the relationship between the various factors affecting OBS and kidney stones could be further investigated.

There are also some limitations to this study, as the data were taken from a nationally representative survey population in the United States, so the conclusions drawn may only be appropriate for the U.S. mainland population, and further research on populations in other parts of the world would be needed to obtain a generalizable conclusion. The research used in this study was primarily in the form of a questionnaire, so there is recall bias. Also, there were many participants with varying degrees of missing information in the raw data, and there was non-response bias. With the data in this study, it is difficult to determine the temporal relationship between antecedents and consequences.

## Conclusion

In conclusion, our study found a negative association between OBS and the prevalence of kidney stones. Higher OBS, indicating more antioxidant exposure than pro-oxidant exposure in diet and lifestyle, was also associated with a lower risk of kidney stone prevalence. This finding suggests that antioxidant diets and lifestyles are beneficial in reducing the incidence of kidney stones, improving people’s quality of life, and reducing the disease burden.

## Data Availability

The data used in this study are publicly available from the National Centers for Health Statistics (NCHS), a branch of the Centers for Disease Control and Prevention (CDC). The website link is: https://www.cdc.gov/nchs/nhanes/index.htm.
